# Prevalence of Bovine Viral Diarrhea Virus Infection in Japan: 2000–2019

**DOI:** 10.3389/fvets.2021.667933

**Published:** 2021-04-30

**Authors:** Motoshi Tajima

**Affiliations:** Department of Large Animal Clinical Sciences, School of Veterinary Medicine, Rakuno Gakuen University, Ebetsu, Japan

**Keywords:** bovine viral diarrhea, BVD, control, prevalence, vaccination

## Introduction

Nearly 4 million cattle are fed in Japan as dairy and beef cattle. More than 50% of dairy cattle (800,000 cows) and 20% of beef cattle (500,000) are fed in Hokkaido, Japan. Young and adult cows in addition to dairy products are delivered throughout Japan from Hokkaido. Moreover, many dairy cows are brought to Hokkaido as calves, and return to their home farm when pregnant. Vaccinations for some diseases are compulsory to prevent infectious diseases when cows move feeding places and particularly when they are introduced to common grazing farms.

Twenty-seven ruminant, equine, swine, avian, and bee diseases are designated as regulated domestic animal infectious diseases (1) as is paratuberculosis (Johne's disease; JD). Another 71 diseases in domestic animals have been monitored as non-regulated diseases since 1998 (2). Bovine viral diarrhea (BVD) and enzootic bovine leukemia (EBL) are non-regulated diseases. The prevalence of JD has been periodically monitored for a long time, and eradication programs are ongoing and also being developed.

Typical clinical symptoms are exhibited by JD-affected animals, and its spread in farms is a serious issue. In Hokkaido, a compulsory examination for the antibody against *Mycobacterium avium spp. paratuberculosis*, the pathogen of JD, is performed once every 5 years by all farms including dairy and beef cattle. Surveillance for a few decades cannot eradicate JD because the pathogen may remain latent in herds for long periods of time and difficulties are associated with its detection in the early stage of the disease. Although bovine leukemia virus (BLV), the pathogen of EBL, is latent in herds, preventive management is possible.

Bovine viral diarrhea virus (BVDV) is associated with various subclinical to fatal diseases. Persistent infection (PI) has been recognized as a serious threat to the cattle industry. PI animals can then subsequently develop mucosal disease which is often fatal. The immune dysfunction associated with BVDV infection has been associated with bovine respiratory disease complex and hemorrhagic syndrome. These disease syndromes have a serious economic impact on cattle producers.

Vaccines are useful tools for the protection of infectious diseases. BVDV is the only one of the three endemic diseases (BVD, EBL, and JD) that cattle are vaccinated for in Japan. The control of PI by BVDV is a key point for its eradication; however, difficulties are associated with detecting PI animals because not all infected animals exhibit the typical clinical symptoms and fetal infection cannot be estimated. Moreover, it is difficult to discriminate whether the immunization status was achieved by vaccination or infection. An update on BVD in Japan is presented in this review.

## Prevalence

**Figure 1:**
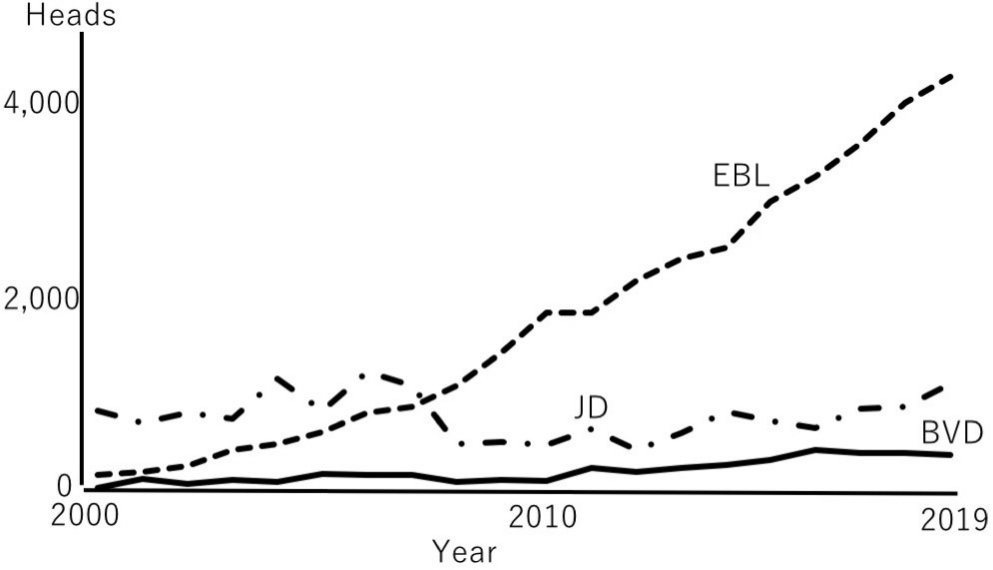
Changes in the number of reported cases of three diseases. Fluctuation of three diseases during 20 years are indicated with different kind of lines, respectively. Plots were based on the reported numbers in MAFF (1, 2). BVD, bovine viral diarrhea; EBL, enzootic bovine leukemia; JD, Johne's disease.

**Figure 2:**
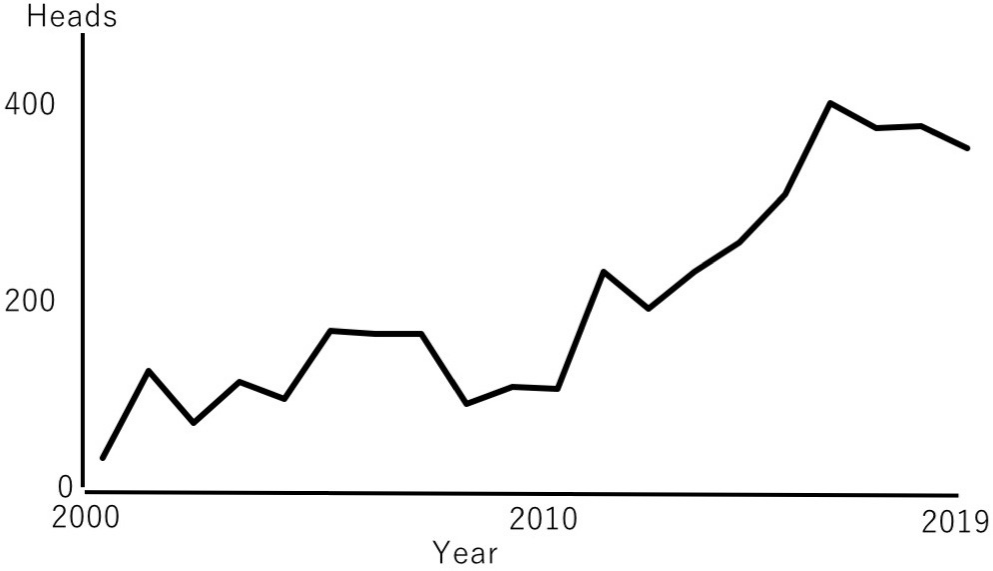
Changes in the number of reported cases of bovine viral diarrhea (BVD). Reported numbers are the same as those in Figure 1. The scale of the Y-axis is adjusted.

The number of reported cases of BVD, JD, and EBL in Japan between 2000 and 2019 varied by pathogen (Figure 1) (1, 2). The number of cattle affected with JD has remained relatively constant every year, ranging between 405 and 1,179 cases in 2012 and 2006, respectively, with a mean of 746 cases per year. The reported number of EBL cases has increased more than those of the JD and BVD, from 161 cases in 2000 to 4,113 cases in 2019, with a mean of 1,693 per year. The number of BVD cases varied between 31 cases in 2000 to 406 in 2016, with a mean of 198 cases per year. The number of BVD cases has also slightly increased in recent years (Figure 2). More than 300 PI cattle per year have been identified since 2015 due to aggressive surveillance, such as the bulk tank milk test, examinations of newborn calves, and regional surveillance (3, 4). Aggressive surveillance may identify BVD and EBL cases without clinical signs. The detection of infected cattle in the early stage of infection may contribute to protection against the spread of the pathogen.

BVDV-1 and -2 (pestivirus A and B) have both been isolated from field cases in Japan. Few sub-genotypes have been recognized in field isolates; however, the HoBi-like virus (pestivirus H, BVDV-3) has not been detected in Japan. Genetic diversity may not vary to the same extent as in European cases (3, 5, 6).

Officially reported BVD cases are PI animals only, which are confirmed cases based on twice positive BVDV results using antigen-ELISA or RT-PCR in over a 3-week interval. Some farmers are reluctant to keep BVDV-positive cows on their own farms for 3 weeks after the first examination, and these cows are culled without confirmation before the second examination. These cases are not recorded in official reports. Acute infections are also not recorded. In long-term infection by acute infection of BVDV, viremia can persist and be defined as persistent positivity for infection with at least 3 weeks in between the tests, and those cases can be classified as PI (7). Thus, these cases are recorded in official reports.

## Vaccine

The first BVDV vaccine introduced in 1973 contained NCP type 1 No. 12 strain. It was initially used to protect against BVD (fatal diarrhea and mucosal disease), particularly for calves and primipara cows. Due to limited information on the pathogenicity of the disease, the accidental production of PI animals occurred following the vaccination of pregnant cows with the modified live virus (MLV). Due to these PI safety issues; the use of vaccine was infrequent. A combination viral respiratory disease MLV vaccine containing bovine herpesvirus 1 (BHV-1) and bovine respiratory syncytial virus (BRSV), along with other viruses was subsequently developed in the 1990's (Table 1). Inactivated vaccine was introduced in Japan in 2002 (Table 1).

**Table 1:** Commercially available vaccines for BVDV in Japan in 2020.

**Vaccine (since)**	**Type**	**BVDV1 strain**	**BVDV2 strain**	**Combined viruses**
5 combi. MLV vaccine (1994)	L	No.12 1a NCP	–	BHV1, BRSV, PI3, AD7
Stockguard^*^ (2002)	K	Singer 1a CP	5912 CP	BHV1, BRSV, PI3
Cattlewin 6 (2005)	K	Nose 1a CP	KZ CP	–
	L	–	–	BHV1, BRSV, PI3, AD7
Bovivac 5 (2011)	K	HK003 1a CP	HK060 CP	BHV1, BRSV, PI3
Bovivac B5 (2014)	K	HK286 1b CP	HK060 CP	BHV1, BRSV, PI3
Calfwin-6 (2014)	L	No.12 1a NCP	KZ12 NCP	BHV1, BRSV, PI3, AD7
Cattlewin 5K (2015)	K	Nose 1a CP	KZ91 CP	BHV1, BRSV, PI3

*L, modified live virus; K, killed virus; BVDV, bovine viral diarrhea virus; BHV1, bovine herpes virus 1; BRSV, bovine respiratory syncytial virus; PI3, bovine parainfluenza 3; AD7, bovine adenovirus type 7*.

*-: not included*

* *: imported vaccine from the USA (original name Pyramid)*.

The development of an inactivated BVDV vaccine and the recognition of respiratory disease complex in herds have expanded the use of the vaccine. However, some veterinarians and farmers have considered that vaccination provides complete PI protection, and sometimes have not been performing BVDV PI detection in vaccinated herds. This has resulted in the failure to identify newborne PI calves. These PI animals could then introduce BVDV into the home herd or other herds.

## Control Trial

A regional voluntary eradication trial has been performed since the 2000's in Japan. The bulk tank milk test to detect PI is conducted to confirm the BVDV status of the farm. In Japan, an estimation of the antibody against BVDV is not an effective tool for the confirmation of infection in farms because the majority of cattle are vaccinated for respiratory disease complex, including BVDV. All examinations for BVDV are based on the detection of the viral gene or antigen using RT-PCR or ELISA. The bulk tank milk tests have been performed on dairy farms for local screening. This test facilitates the detection of PI milking cows. The monitoring of grazing places is effective for detecting PI calves. More recently, in addition to vaccination, calves are often tested for viral infection before being moved to public or private grazing places. Prior to this, vaccination was considered sufficient for protection against BVDV.

There is currently no national eradication program for BVD in Japan. In 2016, the Japanese government recommended projects for BVDV eradication at the herd level as a voluntary program. These projects provided financial support to farms at which PI animals have been detected, which has encouraged farmers to not only clean their farms, but also to identify PI animals by continuously performing virus examinations. It also covers examination fees for all animals in the farm, product loss due to PI, and virus examinations for newborn calves for a few months after the detection of PI animals. However, the cost of the first examination for the detection of PI animals is not included. In contrast to the JD program, this program does not mandate surveillance. However, given that the cost is partially covered, farmers are more likely to participate in the program. The anti-BVD efforts recommended by the government are test-and-cull and vaccination. The necessity of a periodic examination for BVDV PI has recently been recognized.

In summary, BVDV infections as reported by the Japanese government have increased over the last 20 years and peaked in 2016. There is no mandatory Japanese BVDV eradication program. The Japanese government financed a program in 2016 to increase BVDV surveillance to identify PI and to the cull PI animals from the herd. This program has resulted in fewer PI animals being identified in the past few years. BVDV vaccination is a useful tool to aid in BVDV control. A BVDV NCP live virus vaccine was introduced in Japan in 1973. Although infrequently used, this vaccine had safety issues including producing BVDV PI calves. Inactivated BVDV vaccines have been available since 2002. In conclusion, BVDV control in Japan is dependent on voluntary programs using surveillance and vaccination.

## Data Availability Statement

The raw data supporting the conclusions of this article will be made available by the authors, without undue reservation.

## Author Contributions

MT drafted and critically revised the manuscript, contributed to the article, and approved the submitted version.

## Conflict of Interest

The author declares that the research was conducted in the absence of any commercial or financial relationships that could be construed as a potential conflict of interest.
